# Biotechnological Importance of *Torulaspora* *delbrueckii*: From the Obscurity to the Spotlight

**DOI:** 10.3390/jof7090712

**Published:** 2021-08-30

**Authors:** Ticiana Fernandes, Flávia Silva-Sousa, Fábio Pereira, Teresa Rito, Pedro Soares, Ricardo Franco-Duarte, Maria João Sousa

**Affiliations:** 1 CBMA (Centre of Molecular and Environmental Biology), Department of Biology, University of Minho, 4710-057 Braga, Portugal; tixand.tf@gmail.com (T.F.); sousa.s.flavia@gmail.com (F.S.-S.); pg40079@alunos.uminho.pt (F.P.); teresarito@bio.uminho.pt (T.R.); pedrosoares@bio.uminho.pt (P.S.); mjsousa@bio.uminho.pt (M.J.S.); 2 Institute of Science and Innovation for Bio-Sustainability (IB-S), University of Minho, 4710-057 Braga, Portugal

**Keywords:** *Torulaspora delbrueckii*, non-*Saccharomyces*, wine, bread, biotechnology, genomics

## Abstract

*Torulaspora delbrueckii* has attracted interest in recent years, especially due to its biotechnological potential, arising from its flavor- and aroma-enhancing properties when used in wine, beer or bread dough fermentation, as well as from its remarkable resistance to osmotic and freezing stresses. In the present review, genomic, biochemical, and phenotypic features of *T. delbrueckii* are described, comparing them with other species, particularly with the biotechnologically well-established yeast, *Saccharomyces cerevisiae*. We conclude about the aspects that make this yeast a promising biotechnological model to be exploited in a wide range of industries, particularly in wine and bakery. A phylogenetic analysis was also performed, using the core proteome of *T. delbrueckii*, to compare the number of homologous proteins relative to the most closely related species, understanding the phylogenetic placement of this species with robust support. Lastly, the genetic tools available for *T. delbrueckii* improvement are discussed, focusing on adaptive laboratorial evolution and its potential.

## 1. Introduction

Non-*Saccharomyces* yeasts were described for many years as sources of spoilage and contamination, and are also associated with a negative contribution to the organoleptic profile of wines. However, in recent decades, wines produced by some non-*Saccharomyces* yeasts revealed distinct and unique characteristics attracting the attention of many research groups [1]. Improved wines are obtained benefiting from their physiological and metabolic features, which have a positive effect on the wine’s sensorial and chemical properties, namely in terms of sugar and acid consumption, alongside an enhanced aroma complexity through the release of important metabolites [2,3,4]. Within this group of yeasts, *Torulaspora delbrueckii* stands out as one of the most advantageous non-*Saccharomyces* species due to its potential to introduce diversity and multiplicity to the standard wine’s market, currently established by the use of *Saccharomyces cerevisiae* [5]. The rising interest in *T. delbrueckii* is reflected by the number of scientific publications involving this species. According to the Web of Science™ database, between the years 1987 and 2013, an average of eight publications per year were related to the topic *T. delbrueckii* (search queries by title, abstracts and keywords), and this number is continuously growing with a 6-fold increase between 2013 and 2021.

In the present review, we explored available information on the biochemical, genomic and phenotypic features of *T. delbrueckii,* with special emphasis on the aspects that make this yeast a promising biotechnological model to be exploited in a wide range of industries, but particularly in wine and bakery. Genomic, phenotypic, and metabolic characteristics of *T. delbrueckii* were scrutinized to enrich the understanding of this non-*Saccharomyces* yeast, comparing it with the yeast well-established in the market, *S. cerevisiae*. In addition, the importance of applying genetic tools towards *T. delbrueckii* improvement was highlighted.

## 2. Occurrence and General Characteristics

**Figure 1:**
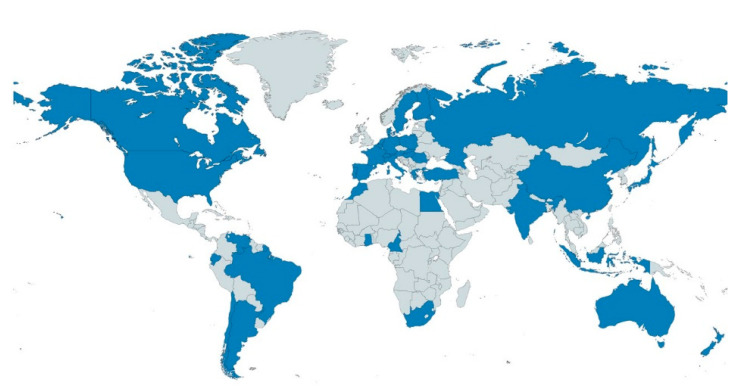
Geographical distribution of *Torulaspora delbrueckii*. Countries in which *T. delbrueckii* isolation was reported are highlighted in blue. Data were collected from Albertin et al. [6], Drumonde-Neves et al. [1] and de Vuyst et al. [17].

Yeasts from the genus *Torulaspora* have been reported in a wide variety of habitats, such as fruits [6], insects [7,8], soils [9], soil invertebrates [10], plants [11,12], seawater [13], spoiled food [6] and malt environments [6], where yeast from other genera like *Saccharomyces* and *Zygosaccharomyces* may also be present [14,15]. Although not considered a human pathogen, the species *T. delbrueckii* can also be found as a clinical isolate [16]. In addition to the diversified isolation substrates, *T. delbrueckii* also presents a worldwide geographical distribution, with reports describing its isolation in 37 countries from the five continents, as shown in Figure 1

Species belonging to the genus *Torulaspora* can reproduce asexually by cell division (budding division) or sexually through asci, containing one to four spherical ascospores, characteristic of ascomycetous yeasts [4,18,19]. Regarding its shape, *Torulaspora* yeasts are mainly discerned by spherical cells (hence the torulu terminology), but also ovoid and ellipsoidal forms, with dimensions of approximately 2–6×3–7 µm, which are smaller than those of *S. cerevisiae*. The sharing of multiple morphological and physiological characteristics between some species has led to a misclassification of some of them. Within the genus *Torulaspora*, four strains presumed to be *T. delbrueckii* were later reclassified into the genera *Debaryomyces* and *Saccharomyces*. Currently, this group includes at least six species: *T. delbrueckii* (anamorph *Candida colliculosa*), *T. franciscae*, *T. pretoriensis*, *T. microellipsoides*, *T. globosa* and *T. maleeae* [20]. Two other species—*T. indica* and *T. quercuum*—have also been proposed to be included in this genus, after the employment of molecular tools to discriminate them [21]. For many years, *T. delbrueckii* was described as a haploid yeast, essentially because of its small cell size and due to the rare detection of tetrads in sporulation media [20]. However, Albertin et al. [6] suggested that this species may be mainly diploid. The reduced size of this yeast is not, in this way, associated with the ploidy level, and may be explained by the fact that *T. delbrueckii* only possesses 16 chromosomes in the diploid phase, instead of the 32 chromosomes found in *S. cerevisiae* diploid yeasts [20]. Given the lack of deep knowledge about the life cycle of *T. delbrueckii*, it is still difficult to design strategies for the biotechnological improvement of *T. delbrueckii* using classical genetic techniques such as those commonly proposed for *S. cerevisiae* [22]. New techniques are, in this way, being explored, as will be detailed further.

The phylogenetic proximity between *T. delbrueckii* and *S. cerevisiae* may contribute to explain why *T. delbrueckii* is one of the non-*Saccharomyces* yeasts suggested to be most promising for use in biotechnological industries, especially the ones using fermentative processes such as wine- or bread making. *T. delbrueckii* was one of the first non-*Saccharomyces* species to be applied commercially in wines, even though only a few species are available in companies’ catalogues: Prelude^TM^, Biodiva^TM^, Zymaflore^®^ Alpha, Vinifer NS^TD^, and Primaflora^®^ VB BIO [4].

## 3. Genomics and Taxonomy

In opposition to the extensive knowledge about *S. cerevisiae* genome, the most thoroughly annotated eukaryotic organism [23], there has been a hinder in progress regarding *T. delbrueckii* genomic characterization, also delaying the understanding of the genomics underlying the unique aptitudes showed by this species, in comparison with other yeasts. The genome of *T. delbrueckii* type strain CBS1146 is organized in eight chromosomes, it is 9.52 Mb long and has a GC content of 41.9% [24]. Recently, our in-depth study [25] analysed publicly available genomes of *T. delbrueckii* strains, improving their annotation and concluding about important intra-strain differences. In terms of genome size, variations between 9.22 Mb and 11.53 Mb were found. This variation corresponds also to a diverse number of protein-coding genes being annotated (between 464 and 503). Interestingly, the similarity obtained when analysing pairwise comparisons between the four tested strains’ genomes was only as high as 99.63%, and in one case was as low as 97.62%. The improved genome annotation obtained in this work allowed to extend this diversity to a particular functional characterization, showing inter-strain differences in proteins related to ATP-synthesis, proton transports, biosynthesis of inositol and resistance to antiviral Brefeldin A. These differences highlight the importance of using different yeast strains in beverages production (and also in other biotechnological applications), improving their quality and diversity.

**Figure 2:**
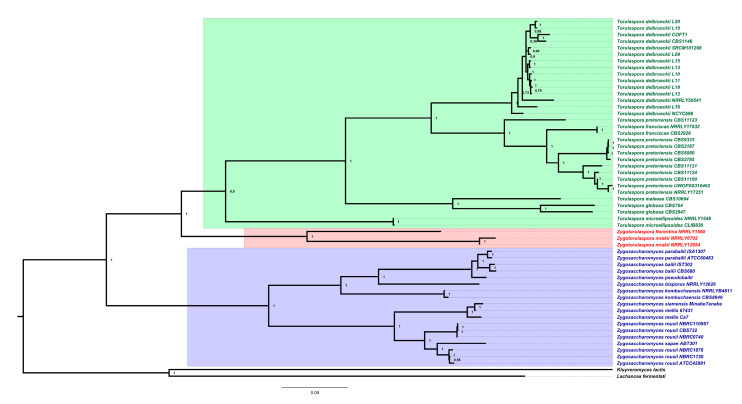
Phylogenetic placement of *Torulaspora delbrueckii,* showing the relationship of 15 strains with publicly available genomes, in relation to its closely related species, chosen from [25]. The phylogenetic reconstruction was obtained using the following parameters: maximum likelihood in IQ-TREE (http://www.iqtree.org, accessed on 28 July 2021), the model of amino acid evolution JTT (Jones–Taylor–Thornton), and four gamma-distributed rates. Homologues were detected for 3820 proteins across the proteome of the 55 fungal species/strains, collected from NCBI. The set of 3820 proteins was aligned and then concatenated for their use in the phylogenetic analysis. These proteins offer a clear high-resolution evolutionary view of the different species, as they are essential proteins beyond the specific biology of the different yeasts. Bootstrapping provided values of 100% for most nodes.

*T. delbrueckii* belongs to the phylum Ascomycota, subphylum Saccharomycotina, class Saccharomycetes, order Saccharomycetales, family Saccharomycetaceae. In our previous work [25] we detailed the *T. delbrueckii* phylogenetic placement in relation to 386 other fungal species/strains, concluding about the proximity between this species and the genera *Zygosaccharomyces* and *Zygotorulaspora*. Our results were in accordance with the work of Shen et al. [26], which showed the phylogenetic reconstruction of more than 300 budding yeasts, even though the *T. delbrueckii* branch was concluded as not being robustly supported. Aiming at elucidating the proximity between the three genera—*Torulaspora, Zygosaccharomyces* and *Zygotorulaspora*—we performed a robust phylogenetic reconstruction, filling this gap with the inclusion of additional genomes publicly available in NCBI. As can be depicted in Figure 2, all the 15 available genomes of *T. delbrueckii* were grouped together in a single isolated clade (highlighted in green in Figure 2), separated from the ones of *T. pretoriensis*, *T. franciscae*, *T. maleae*, *T. globosa* and *T. microellipsoides*. The large branch containing all genomes of the genus *Torulaspora* revealed to be isolated from *Zygotorulaspora* clade (containing species *Zygotorulaspora florentina* and *Zygotorulaspora mrakii*, and highlighted in red in Figure 2). In addition, both these genera—*Torulaspora* and *Zygotorulaspora*—formed an isolated group, separated from the one containing *Zygosaccharomyces* species (highlighted in blue in Figure 2).

## 4. Metabolism

Concerning *T. delbrueckii* fermentative behaviour, no consensus has yet been gathered regarding its fermentative power. Some authors characterized this species as having a good fermentation performance in wines [3,4,14,20]. Bely et al. [14] even categorized *T. delbrueckii* as having a performance 9 to 10% higher when considering other non-*Saccharomyces* yeasts. On the contrary, Belda et al. [27] and Loira et al. [28] concluded that *Torulaspora* spp. have lower fermentative power. Still, Almeida and Pais [29] described similar fermentation ability for *T. delbrueckii* and *S. cerevisiae* strains in bread dough. These observations could support the idea of a strain dependent profile with respect to the fermentative capacity of this species, which is also supported by our unpublished data showing a heterogenous performance when analyzing a collection of *T. delbrueckii* strains.

*T. delbrueckii* presents poor fructose and glucose consumption under conditions of high ethanol and moderate acetic acid concentrations, that can be present in stuck wine fermentations, although it can survive in this environment. This behavior has been associated with the sensitivity of its hexose transport to the inhibitory effect of ethanol [30,31]. To address this limitation, a hybrid strain (F1-11) was constructed by Santos et al. [31] combining the advantageous characteristics of high tolerance to both ethanol and acetic acid of *T. delbrueckii,* and the high hexose consumption of *S. cerevisiae*. This hybrid exhibited a hexose consumption comparable to the one of the *S. cerevisiae* and revealed improved resistance to ethanol and acetic acid, presenting lower cell death rates.

Comparatively, both *T. delbrueckii* and *S. cerevisiae* species behave quite particularly regarding oxygen availability. As the oxygen feed rate decreases, *S. cerevisiae* is the first yeast to switch to a respiro-fermentative metabolism, thus exhibiting lower biomass yields at reduced amounts of oxygen, in comparison to *T. delbrueckii*, which is able to maintain full respiration under these conditions, translating into a lower fermentation strength and a slower growth rate [32]. This occurrence could be less favorable in a winemaking environment since wine production is usually performed under strictly anaerobic conditions (e.g., white, and sparkling wine), or in the presence of very low oxygen concentrations (e.g., red wines) [33].

Even though *T. delbrueckii* possesses lower tolerance to low-oxygen conditions [14,34], its metabolism is usually associated with several positive characteristics, mainly regarding the wine industry, related to high osmotic and sulphur dioxide resistance [23,24,25,26,27,28,35], enhanced capacity for biotransformation of terpenes [28,36,37], or high competence to produce lactic and succinic acids [28,36].

The volatile acidity associated with wine is mainly due to the presence of acetic acid which, above a threshold of 0.8 g/L is considered as negatively affecting the quality of the product, contributing to a vinegar character. Regarding acetic acid production, *T. delbrueckii* presents an advantage towards *S. cerevisiae*, since it typically produces lower levels. In fact, according to Bely et al. [14], it originated between 0.27 g/L and 0.56 g/L of acetic acid, even in high-sugar fermentations, while *S. cerevisiae* produced amounts ranging between 1.0 and 1.17 g/L. Values reported for *S. cerevisiae* can be even higher under some conditions, reaching up to 1.8 g/L [14,20] and Paraggio et al. [38] reported acetic acid production as a strain-dependent feature of this yeast. Other works have reported even lower values of acetic acid production by *T. delbrueckii*, ranging from 0.14 to 0.28 g/L [4,14]. *T. delbrueckii* is also a lower producer of acetaldehyde, in comparison with *S. cerevisiae*, which is an important advantage, since concentrations above 125 mg/L of acetaldehyde pertain to a negative effect on wine’s flavor, often being described as oxidized. In red wines, acetaldehyde is, on the other hand, described as being of interest to produce vitisin B, a red stable phenol compound [4]. In fact, vitisin B belongs to the group of the most stable anthocyanins, which are normally used as a strategy to increase the intensity of wines’ red color. Nevertheless, beyond this strategy, color perception can be increased through a reduction in the pH [5]. Regarding malic acid degradation, some contrasting reports characterize *T. delbrueckii* as consuming, for one side, between 20–25% of this acid [27,39], but on the other side as having no consumption at all [28]. Malic acid degradation seems to be, in this way, a strain-dependent feature, as also concluded by Du Plessis et al. [40]. In a similar way to malic acid, citric acid is an organic acid produced through the Tricarboxylic Acid Cycle (TCA) pathway, standing out as a pleasant citrus-like taste contributor to wines’ aroma profile. Liu et al. [41] carried out pure fermentation of *T. delbrueckii* and *S. cerevisiae* and observed similarities between both species in terms of production of this acid, reaching concentrations of 2.18–2.36 g/L and 2.23 g/L, respectively.

Another favorable characteristic associated with the use of *T. delbrueckii* is its higher production of succinic acid, in comparison with *S. cerevisiae*, being able to reach concentrations above 1 g/L depending on the oxygen availability during fermentation [41,42]. In fact, Puertas et al. [43] reported productions by *T. delbrueckii* between 0.84 g/L and 1.11 g/L, while in the same study *S. cerevisiae* only reached maximum values of 0.65 g/L. However, Franco-Duarte et al. [44] obtained maximum concentrations of 1.13 g/L using natural isolates of *S. cerevisiae*. Alongside, in comparison with *S. cerevisiae*, *T. delbrueckii* generates amounts of mannoproteins up to 25% higher (expressed as mannose concentration) that are released from the yeast cell during alcoholic fermentation and during the ageing processes, and contribute to the chemical stabilization of white wines [4,5]. A similar study was performed by Domizio et al. [45], where *T. delbrueckii* revealed up to 50% more polysaccharides, in pure fermentations, than the *S. cerevisiae* controls.

Oenological importance of glycerol stands out by its enhancement of softness and viscosity of wine [4,5]. Wines fermented by non-*Saccharomyces* yeasts, particularly *T. delbrueckii*, are recognized to result in slightly higher glycerol yields than those fermented by *S. cerevisiae*, in concentrations ranging between 1 g/L [46] and 10.5 g/L [47] for *T. delbrueckii*, while *S. cerevisiae* revealed maximum concentrations of 9.1 g/L (depending on the wine style). Beyond glycerol production, *T. delbrueckii* is usually described as a low ethanol producer (40.6–72.7 g/L [48]) in comparison to traditional fermentations carried out by *S. cerevisiae* [4,7]. These concentrations are in a much lower range of values than the one obtained with *S. cerevisiae* strains—103 to 121 g/L [49]. In addition, the great mouthfeel traits [14] reinforce the wide potential of *T. delbrueckii* in winemaking [7,28,50]. Table 1 reviews experimental results obtained regarding the most relevant fermentation parameters towards wines’ organoleptic profile, comparing *T. delbrueckii* and *S. cerevisiae*.

Regarding higher alcohols, several authors presented contradictory results related to high and low levels of these compounds as produced by *T. delbrueckii* [2,23,51], leading to conclude that this characteristic is, once again, strain-dependent. Particularizing for nitrogen metabolism, Bely et al. [14] observed higher residual nitrogen levels in pure fermentation with *T. delbrueckii*, in comparison to those obtained with *S. cerevisiae* alone. This discrepancy may be due to the fact that this species is less demanding for amino acids.

## 5. Biotechnological Importance of *T. delbrueckii*

### 5.1. Bread Industry

Bread making is a practice that has long been discovered and has been the subject of much progress. In more recent years, developments in bread making have been increasingly focused on the enhancement and diversification of the sensory pleasures of taste, texture, and appearance of the final product [19]. The degradation of the dough carbohydrates (namely fructose, glucose, sucrose and maltose) present in the flour, or even wittingly added, is carried out by yeasts, resulting in the release of carbon dioxide and ethanol, produced through glycolysis and posterior pyruvate decarboxylation and reduction [17,19,30]. Carbon dioxide is responsible for the dough leavening, while most of the ethanol evaporates during the baking process. However, the latter also plays an important role in the properties of the dough [17]. The choice of the appropriate yeast is usually based on (i) good fermentative power which could be translated into its ability to leaven the dough; (ii) capacity to use different carbon sources; and (iii) tolerance to stressful conditions, namely, osmotic, and freezing stresses [30,52,53]. *S. cerevisiae* strains have been domesticated and optimized for baking applications and are usually the manufacturer’s required yeast for the baking industry. This species efficiently uses maltose as a source of energy, as opposed to *Candida humilis* and *Kazachstania exigua* which, according to de Vuyst et al. [17], are sourdough-specific maltose-negative yeasts. *S. cerevisiae* is commonly implemented as a leavening agent, becoming an alternative to sourdough (extensively used for years) particularly in rapid and industrial-scale bread productions [17]. However, *T. delbrueckii* is being pointed out as an alternative to *S. cerevisiae* in this industry, mainly due to its high osmotic and freeze-thawing resistance, showing improvement of the quality of the bakery products [29,30]. Experiments conducted by Almeida and Pais [29] demonstrated greater leavening activity in lean and frozen dough for *T. delbrueckii* strains, comparing to *S. cerevisiae*, as the traditional yeast was more prone to suffer from freeze damage during the storage of the doughs. Apart from this feature, *T. delbrueckii* strains displayed rapid growth, a more rapid response when exposed to hyperosmotic conditions, and high biomass production accompanied with sweet properties (associated with the release of aromatic compounds). These observations were later confirmed by Hernandez-Lopez, Prieto and Randez-Gil [54]. Due to its osmotolerant properties, *T. delbrueckii* has already been used in the bakery industry in Japan, for the production of sweet breads and pastries [55].

Co-cultures using *S. cerevisiae* and *T. delbrueckii* species enhanced bread quality with superior aroma and improved sensorial attributes, with 47 volatile compounds—predominately alcohols, aldehydes, and esters—being identified in the bread crumb leavened with both yeasts [19]. Wahyono et al. [19] highlighted some properties of the resulting mixed bread which, using a radar plot, rated within a range of 4.73–5.57 from a total of 7 points, such as acceptability, enhanced flavor, mouthfeel, and color, in comparison with *S. cerevisiae* single cultures, which recorded within 4.07–5.71 range in the same radar plot.

### 5.2. Production of Fermented Beverages

In recent years, researchers worldwide have been paying particular attention to *T. delbrueckii* exploitation to improve wines organoleptic final profile and quality. As referred above, its physiological and metabolic properties revealed positive effects in wines characteristics towards acids and sugar consumption, but also an enhancement of the aroma complexity through the production of important metabolites [2,3,4,23,56,57,58,59]. During wine fermentation, higher alcohols (also termed fused alcohols) and esters contribute 30 to 80% to the aroma profiles of wine, being the two most relevant groups of metabolites [59]. Isobutanol, phenyl ethanol and isoamyl alcohol are the main fusel alcohols reported to contribute to the wine’s scent in concentrations ranging from 1.41 mg/L to 9.2 mg/L [60]. According to Ebeler [61], yields of this type of metabolites can achieve 140–420 mg/L, but concentrations over 300 mg/L contribute negatively to the aroma quality. Besides fusel alcohols, the aromatic matrix of wine is composed of esters, which are by-products of yeasts metabolism during malolactic fermentation, ageing and, most relevant in this context, alcoholic fermentation. These molecules reach maximum values when yeasts achieve the stationary growth phase [62], as its production by *T. delbrueckii* is a strain-dependent feature [60]. Two main esters classes are present in wine: the ethyl esters and the acetate esters. The contribution of the latter encompasses desirable floral and fruity sensory properties in wine, contributing about 75% to the flavor profile [60,61,62]. However, as stated in Belda et al. [62], wines holding concentrations of ethyl acetate higher than 90 mg/L are considered to be faulty. Other important metabolites are fatty acids, which are detected in alcoholic beverages as mainly straight-chain and saturated molecules, with palmitoleic acid considered the most relevant unsaturated fatty acid. Besides these, fatty acids with different chain lengths are part of the wine’s matrix but prevail in small amounts, which makes them not so significant as the previous ones [61].

In order to respond to the consumers’ demands for wines with low content of ethanol, alongside with obtaining innovative and differentiated wine’s profiles, non-*Saccharomyces* yeasts stand out as the organisms per excellence to achieve a reduction in initial ethanol content by about 1–2% (*v*/*v*), having into account the used species and the conditions in which fermentations are performed [5]. Although the production of wines using *T. delbrueckii* cultures can be more expensive and time-consuming, in comparison to those produced with *S. cerevisiae* [33], the fermentation of grape juice with *T. delbrueckii* tends to originate wines with lower content of alcohol and, at the same time, with higher levels of glycerol. These properties are particularly advantageous for full-bodied and well-structured red wines, obtained from grapes with increased maturity [42,63]. Global warming is a concerning and alarming issue at different levels, also with regard to viniculture, since this situation has an impact on the accelerated ripening of grapes promoting a faster increase in their sugar content which, ultimately, result in the production of wines with increased alcohol content [63,64]. Thus, the search for new yeasts that completely consume sugars and have the ability to both decrease the final ethanol yields and increase the glycerol concentrations is imperative for the construction of organoleptically improved wines, as an alternative to the standardization of the food industry. The variation found between yeast strains plays a very important role to address this issue. In many cases, producers choose to mix different strains to improve the quality of the beverages, and to balance their aromatic and fermentative profile.

Co-cultures of *T. delbrueckii* and *S. cerevisiae*, in synthetic grape must medium show that from the moment *S. cerevisiae* is inoculated, the viability of *T. delbrueckii* decreases even if it is at higher concentrations than the former. This was confirmed by Taillandier et al. [65] who used an inoculum of *T. delbrueckii* twenty times higher than *S. cerevisiae* and observed growth inhibition of the first yeast. As a valid explanation of the phenomenon, the authors pointed to the possible metabolite release by *S. cerevisiae*, excluding substrate competition and cell-to-cell contact mechanism as probable causes. On the other hand, Azzolini et al. [3] demonstrated clear distinct aroma patterns of sequential fermentations of *T. delbrueckii* and *S. cerevisiae,* and of *S. cerevisiae* individual inoculations. The authors detected (i) improvements in the analytical profile and flavor complexity of wines; (ii) freshness and acidity sensory features; (iii) floral and more differentiated wines. Thus, the introduction of non-*Saccharomyces* species in the manufacturing of wines is a useful tool to modulate the organoleptic profile of wines and include innovative and differentiated styles compared to the standardized wines already present in the market.

The dominance ratio of T. delbrueckii when inoculated in fresh must depends on numerous factors, from the inoculum size, quantity, and type of viable wild microorganism initially present in the must, fermentation stage, sugar and ethanol concentrations, killer and killer sensitivity phenotype of the inoculated yeast, the concentration of SO_2_ and copper, pesticides potentially present in grapes, among many other [12]. As a result, under the conditions usually present in grape must fermentations, *T. delbrueckii* initial population growth is high, becoming a protagonist at an earlier stage of the process [23]. Since sugar-rich musts originate high levels of ethanol, in the latter stages, slower fermentation rates and increased cell death of this yeast are observed, probably as a result of its lower resistance to high ethanol concentrations in comparison with *S. cerevisiae* and of its higher hexose transport sensitivity [31]. As a consequence, the process may cease prematurely or become sluggish unless a more resistant yeast is added in a co-inoculation or sequential process, as aforementioned [15].

In addition to wine, *T. delbrueckii* can be explored in the dynamics of other beverages. One example with a great economic value and one of the most popular drinks around the world is beer. The wort ingredients—composed of malted cereals, hops, and fresh water—are transformed not only in alcohol but additionally into organoleptic compounds released by the yeasts, such as aldehydes, higher alcohols, esters, carbonyl compounds, organic acids, and terpenic substances, giving identity to the beer as the final product. However, just like wine, consumers have been switching from classic style beer to new beer-blended beverages as they look for innovative aroma palates. Since this can be achieved by using different yeast strains, the search for new yeasts, particularly in the non-*Saccharomyces* group including *T. delbrueckii*, has increased [63,66,67]. Three studies have reported the aromatic profile of beers influenced by the use of *T. delbrueckii* strains in the brewing process [35,68,69]. These yeasts displayed the ability to transform hop aroma terpenoids and enhance ethyl hexanoate and ethyl octanoate levels.

One particular application of *T. delbrueckii*, only superficially explored, is in the mezcal fermenting process. The use of this species in this process revealed an increase in the levels of *β*-fructofuranosidase enzymes with fructosyltransferase activity [70], and also high levels of phenyl acetate [71]. Similarly to other applications, mezcal fermentations benefits from a mixed inoculum of *S. cerevisiae* and *T. delbrueckii*, to obtain a balanced aromatic and fermentative profile. Furthermore, in cider production, monoculture fermentations using *T. delbrueckii* strains showed to produce more diverse volatile compounds than with *S. cerevisiae* strains [72]. In another study from 2019, Lorenzini et al. [73] tested several *Saccharomyces* and non-*Saccharomyces* yeasts for their capacity to ferment apple juice and to influence the volatile compound production in cider fermentations. Among non-*Saccharomyces* yeasts, *T. delbrueckii* was the greatest producer of ethyl decanoate and ethyl hexanoate, key aroma compounds in cider production, conferring fruity aromas.

The main fermented beverages in which *T. delbrueckii* is employed are reviewed in Table 2.

### 5.3. Other Food Applications

The reported versatility of *T. delbrueckii* makes it a remarkable asset to be explored, not only for bread and fermented beverages purposes, but also in other diverse food products (Table 3). One example is the production of chocolate in which yeasts play a key role in flavour development, as the quality of chocolate is reduced if the cocoa fermentation process is conducted without these microorganisms [76]. This importance is reinforced by Visitin et al. [77] by showing the involvement of *T. delbrueckii* in the fermentation of cocoa beans (*Theobroma cacao* [76]) to produce chocolate, despite not yet being standard in this industry. Authors showed that through a combination with *S. cerevisiae*, modifications on the analytical profile of the chocolate are obtained. Moreover, differences in the samples obtained from *S. cerevisiae* and *T. delbrueckii* inoculated chocolate had a significant impact on the consumers’ perception of the final product, mentioned by some as fruitier. Therefore, the use of this unconventional yeast resulted in a positive contribution to the development of the chocolate’s final aroma. In addition, *T. delbrueckii* can also be explored in the cheese industry, benefiting from its tolerance to low temperatures, low pH, high salt concentrations and low water activity [78]. Andrade et al. [79] produced cheese from fermented milk, with the aim of evaluating the impact of *T. delbrueckii* (in mixed or pure inocula) on cheese production, detecting a slow consumption of lactose which can be translated into a reduced *β*-galactosidase activity, as stated by the authors.

Another highly sought product in the food market is honey. This is produced by honeybees (namely *Apis mellifera*) and is a natural source of fermentable sugar ready to be used by fermentative yeasts [7]. Barry et al. [7] were able to isolate two *T. delbrueckii* strains directly from the microbiome of a honeybee and use them to ferment honey, obtaining interesting results to be applied at an industrial scale, especially when in combination with a *S. cerevisiae* champagne strain. The list of applications of *T. delbrueckii* can be extended also to the fermentation of olives, with Psani and Kotzekidou [81] reporting good outcomes from the exploitation of this species, such as being able to hydrolyse olive oil and tributyrin, alongside the capacity of *T. delbrueckii* cultures to inhibit foodborne pathogens such as *Listeria monocytogenes*, *Bacillus cereus*, and *Salmonella typhimurium*. However, the authors also detected a strong growth inhibition of *T. delbrueckii* by the assimilation of oleuropein at yields greater than 0.5% (*w*/*v*). *T. delbrueckii* can also be employed in the fermentation of coffee beans, one of the most popular consumed beverages. Coffee fermentation occurs naturally, however, the use of yeast as a starter culture was shown to improve coffee flavour and aroma. Da Mota et al. [82] showed that *T. delbrueckii* inoculation exhibited the best performance in natural coffee compared to *S. cerevisiae* and to the control (without inoculation), by positively improving the sensorial quality of the final product. Nevertheless, other authors also reported that the performance of *T. delbrueckii* may vary according to coffee varieties, production regions, processing methods, and microbial species naturally present in the fruit [83,84,85]. In general, the use of *T. delbrueckii* during coffee fermentation can result in coffees with distinct aromas and flavours that increase the possibility of producing speciality coffees, adding value to the product. In addition to the food applications mentioned so far, the yeast *T. delbrueckii* has also been proposed as a biocontrol agent against spoilage organisms improving the product quality and reducing the use of chemical preservatives to control food spoilage. Simonin et al. [50] reported the successful implantation of the *T. delbrueckii* strain as an alternative to sulphiting without compromising the fermentation kinetics in two Burgundian wineries. Furthermore, Al-Qaysi et al. [4] also revealed a high inhibitory effect of *T. delbrueckii* against the plant pathogens *Fusarium oxysporium*, *Sclerotinia sclerotiorum*, and *Macrophomina phaseolina*, inhibiting mycelial growth in 55.3%, 66.2%, and 31.11%, respectively.

## 6. Genetic Tools Employed towards *T. delbrueckii* Improvement

The prompt progress of genetics, engineering and biology fields enhanced the continuous search for yeasts with improved traits and phenotypes in order to expand their abilities to be further implemented in the most diverse research areas or likewise for commercialization. Yeasts are an advantageous research model due to the easiness with which genes can be manipulated, in particular, recurring deletions, insertions or modifications under controlled conditions. The combination of classical genetic approaches, transformation methods, and DNA sequencing techniques have helped in the molecular characterization of yeasts over the years. In order to apply these techniques, a deep understanding of yeast’s biological diversity is mandatory, to explore the different metabolic pathways and to incorporate the great degree of biological inter-strain diversity.

Regarding genetic tools development to manipulate *T. delbrueckii* strains, already in 1989, Compagno et al. [86] showed for the first time that a 2 *µ*m vector could be maintained and replicated in *T. delbrueckii*. Later on, the discovery of an endogenous circular plasmid pTD1 (4.8 kbp) from *T. delbrueckii* strain CBS1090 was reported [78]. Over the past few years, several attempts successfully identified, cloned, deleted or expressed several genes in *T. delbrueckii* [87,88,89,90,91]. This knowledge opened the way to the improvement of *T. delbrueckii* metabolism and to the development of new genetic tools. In recent years, CRISPR/Cas9 (Clustered Regularly Interspaced Short Palindromic Repeats/associated protein 9) revolutionized research as a gene-based editing mechanism allowing the selective manipulation of DNA [92,93]. Although there is yet no current employment of CRISPR towards *T. delbrueckii*, this engineering tool has been extensively used for other non-conventional yeasts such as *Yarrowia lipolytica, Pichia pastoris*, *Komagataella phaffii, Kluyveromyces lactis, Kluyveromyces marxianus* [94], *Schizosaccharomyces pombe, Candida albicans* and *Candida glabrata,* and for the conventional yeast *S. cerevisiae* [92,93,94,95,96,97].

Besides the versatility of *T. delbrueckii* species, some limitations have been hampering its wide biotechnological use, such as the limited ethanol resistance of some strains during wine fermentation, which ranges from 7.4% (*v*/*v*) [14] to 9% (*v*/*v*) [36], in addition to the fact that Belda et al. [27] indicate decreases of cells viability already when ethanol levels exceed 8% (*v*/*v*). In this context, some strategies have been proposed in this species in order to improve phenotypes with a biotechnological impact, such as random mutagenesis, sexual hybridization, bioprospecting and metabolic engineering [67,96] to expand the boundaries of *T. delbrueckii’s* biotechnological use. The use of Genetically Modified Organisms (GMOs) involves a certain ambiguity and divergence of opinions, and their implementation relies on the legislation in force in each country [97,98]. Currently, the unacceptance of the use of GMOs by the International Organization of Vine and Wine (OIV) makes the products obtained by New Genomic Techniques (NGTs), such as CRISP, unsuitable for commercialization in the European Union (EU) [99,100]. In the EU, Regulation (EC) No. 1829/2003 (article 1) and Regulation No. 1830/2003 on genetically modified food aim to ensure the achievement of a high level of protection to environmental, human, and animal health, alongside the tracing and labelling of genetically modified products, respectively. Although the Directive 2001/18/EC (in particular, article 23) allows the Member States to deliberate whether they restrict or prohibit the cultivation of genetically modified crops in their territory, in 2018, the European Court of Justice clarified that organisms modified by site-directed mutagenesis like CRISPR would be included in the definition of a GMO [101]. On the other hand, the United States has no specific federal law aimed at regulating GMOs, with the country being considered a major developer and marketer of genetically modified crops [102,103,104]. Only two registered and approved genetically modified wine yeasts are currently used in Canada, the USA, and Moldova [105,106,107].

In this context, adaptive laboratory evolution (ALE) emerges as a successful non-genetically modified approach to improve yeast features. According to Dragosits and Mattanovich [108], ALE stands as a powerful tool to study the evolutionary phenomena occurring in microorganisms in controlled laboratory environments, being already performed 100 years ago by William Dallinger. However, these sorts of studies have been explored frequently only in the past 25 years, employing *S. cerevisiae* and *Escherichia coli* as the main models. ALE helps to gain insight into the basic molecular evolution mechanisms where microorganisms are usually inoculated for long-term adaptation (lasting for weeks or months) under specific selective stress conditions [108]. During the design of ALE experiments, several parameters need to be taken into account such as (i) the genetic diversity sources, which includes natural mutations, UV or chemical mutagenesis, or mating; (ii) the selective pressure that could be constant, increased or intermittent over time; and (iii) the cultivation strategies which can be diverse but the most used ones are batch cultivations (performed as sequential serial transfers) or continuous cultures (where the conditions are kept constant and the limitation of one nutritional component is standard) [108]. When exposed to these environments, different types of mutations are identified, such as single-nucleotide polymorphisms (SNPs), transposable elements, small scale insertions and deletions (indels), large scale amplifications or deletions, which contribute to certain changes at the gene regulation and fitness levels, giving rise to improved phenotypes [108]. Several studies performing adaptive evolution for *S. cerevisiae* and focusing on nutritional adaptation, reported increased biomass yields and decreased fermentative performance in glucose-limited selection environments [108,109]. Jansen et al. [110] also observed modifications in the morphology of the cells, alongside with higher affinity for glucose. Regarding stress limitations, Dhar et al. [111] described that increases in salt tolerance by ALE were related to increases in the genome size and changes in the expression of several genes. Other studies focused on improvements of ethanol tolerance by *S. cerevisiae,* have detected a higher behavioral variability of the evolved clones in comparison with the parental strain, when levels of ethanol in the medium were increased from 6 to 8% (*v*/*v*) [112], as well as higher osmotic and temperature tolerances, in comparison with the original strain [113]. Avrahami-Moyal et al. [114] suggested that the stability of the cell wall is an essential factor, and that mutations in the translational regulator *SSD1* and *UTH1* are responsible for the ethanol tolerance of this species. This type of study benefits from the wide inter- and even intra-strain diversity observed, manifested also in genomic differences during the maintenance of microbial cultures in different environmental conditions, and associated with adaptive microevolutionary changes observed even within the descendants of the same strain [115]. When considering *K. marxianus*, strains with higher growth rates were obtained under stressful high ethanol environments [116], within the range of 7–10% (*v*/*v*) of ethanol [117]. In a similar way, a recent study and, to the extent of our knowledge, the only report applying this technique to the generation of *T. delbrueckii* evolved strains, was conducted by Catrielo et al. [96]. The authors efficiently obtained evolved clones of *T. delbrueckii* with improved growth kinetic parameters, increased ethanol resistance, variants capable of tolerating ethanol concentrations of 11.5% (*v*/*v*), and additional higher resistance to SO_2_, in comparison with the original strain. In addition to these features, co-inoculations with the evolved *T. delbrueckii* and *S. cerevisiae* clones were performed, evidencing improved contribution effects to the aromatic profile of wine, with particular emphasis to 2-ethylhexanol, total alcohol levels, total aldehydes, total sulphur compounds and total phenolic derivates with significant differences to the control fermentation, designated only by *S. cerevisiae* evolved clone. Besides this parameter, other studies conducted with *S. cerevisiae* model reported increases in copper resistance mediated by increased expression of *CUP1*, and decreased levels of the transporters *CTR2* and *CCC2* in the evolved strain, together with lower activity of antioxidant enzymes [118].

## 7. Conclusions

*S. cerevisiae* is still the yeast of choice in different biotechnological areas, namely in the wine and bakery industries. However, many studies have pointed out the great interest of *T. delbrueckii* for industrial exploitation. Now, a higher number of studies are necessary to assess the phenotypic, metabolic and genomic landscape of this species, and, especially, to address strain similarities/dissimilarities with great importance to allow its more extensive and rational exploitation, as detailed in this review. With advances recently obtained in the genome characterization and annotation of *T. delbrueckii*, the elucidation of the molecular bases that underlay this species’ specific traits will start to be revealed. This will allow researchers to highlight particular advantages of this species that have caught the attention of the bread and wine industries, and to overcome problems that make it less advantageous in relation to *S. cerevisiae*.

## Figures and Tables

**Table 1:** Comparison between *Torulaspora delbrueckii* and *Saccharomyces cerevisiae* concerning fermentation parameters quantified at the end of fermentation process with relevance in wine organoleptic profiles.

Product	*Torulaspora Delbrueckii*	*Saccharomyces Cerevisiae*	Notes	References
Acetic acid	0.27–0.56 g/L	1.0–1.17 g/L	Key signature in volatile acidity of wines	[14,20]
Malic acid	Consumption between 10.5–25%		Whether degradation or production is desirable depends on the must characteristics.	[23,39,48]
Citric acid	2.18–2.36 g/L	2.23 g/L	Citrus-like taste	[41]
Succinic acid	0.84–1.11 g/L	Maximum of 0.65 g/L	Minor acid in the overall wine acidity, although the combination with one molecule of ethanol creates the ester mono-ethyl succinate, responsible for a mild, fruity aroma	[43]
	-	Maximum of 1.13 g/L		[44]
Mannoproteins	*T. delbrueckii* produces 25% more than *S. cerevisiae*		Released during fermentation or ageing processes	[23]
Polysaccharides	*T. delbrueckii* releases 50% more than *S. cerevisiae*		Increases the quality of mouthfeel properties	[45]
Glycerol	1–10.5 g/L	Maximum of 9.1 g/L	Smoothness and viscosity features	[46,47]
Ethanol	40.6–72.68 g/L	103–121 g/L		[48,49]

**Table 2:** *Torulaspora delbrueckii*’s applications in fermented beverages.

Beverages Applications	Used Substrate	Advantages	Disadvantages	References
Beer	Wort	High tolerance to hop compounds; good flavor-forming properties	Low sugar utilization	[35,66,68,69]
Mezcal	Agave juice ^†^	Rich in volatile compounds; acceptable in sensory tests	Low performance	[67,70,71]
Tequila	Agave juice *	Positive influence on the final sensory profile	–	[74]
Cider	Apple juice ^†^	Great production of ethyl decanoate and ethyl hexanoate	Low performance; negligible amounts of acetate esters	[72,73]
Mead	Honey sugar	Good fermentation ability; Good sensory features	Grassy flavor	[7]
Soy alcoholic beverage	Soy whey	Enrich aroma profiles: high levels of ethyl decanoate and ethyl hexanoate; metabolize hexanal;	–	[75]

* Specifically from *Agave tequilana*; ^†^ sterile.

**Table 3:** *T. delbrueckii* industrial food applications.

Food Applications	Used Substrate	Advantages	Disadvantages	References
Chocolate	Cocoa beans	Good flavor quality of cocoa and, therefore, the chocolate	Expedite in mixed fermentations with *S. cerevisiae*	[77]
Cheese	Cheese	Varied aromatic properties	Unable to inhibit pathogenic bacteria; low β-glucosidase activity	[79,80]
Honey	Honey sugar	Rapidly ferment sugar	Large-scale productions only in combination with *S. cerevisiae*	[7]
Olive oil	Black olives	Easy hydrolyzation of olive oil	Growth inhibition at concentrations higher than 0.5% (*w*/*v*) of oleuropein	[81]
Coffee	Coffee cherries	Improve coffee’s sensorial quality	Pronounced astringency depending on the coffee variety	[82,83]
Bio-protection	–	Reduction in the use of chemical preservatives to control food spoilage	–	[50,84]
